# Treat me nice! –a cross-sectional study examining support during the first year in the emergency medical services

**DOI:** 10.1186/s13049-018-0561-7

**Published:** 2018-11-06

**Authors:** Anna Hörberg, Susanne Kalén, Maria Jirwe, Max Scheja, Veronica Lindström

**Affiliations:** 1 Karolinska Institutet, Department of Clinical Science and Education, Södersjukhuset, Academic EMS, Stockholm, Sweden; 2 Karolinska Institutet, Department of Clinical Sciences and Education, Södersjukhuset, Stockholm City Council, Stockholm, Sweden; 3 Karolinska Institutet, Department of Neurobiology Care Sciences and Society, Division of Nursing, Stockholm, Sweden; 4 Department of Education, Stockholm University, Stockholm, Sweden; 5 Department of Neurobiology Care Sciences and Society, Division of Nursing, Academic EMS Stockholm, Stockholm, Sweden

**Keywords:** Education nursing, Emergency medical services, Nurses, Professional role, Professional development, Support

## Abstract

**Background:**

Working in the emergency medical service (EMS) can be extremely varying and sometimes physically and psychologically demanding. Being new in this context can be a great challenge. This study aim to describe what ambulance nurses consider to be important support during the first year in the EMS.

**Methods:**

Three hundred and eighty-nine eligible participants that had graduated from the prehospital emergency care program were identified via university registrations office in Sweden. The eligible participants received a study specific questionnaire via mail consisting of 70 statements about support during the first year. The perceived importance of each statement were graded on a 7-point Likert scale. The gradings were analysed using descriptive statistics and frequencies, mean and SD were calculated.

**Results:**

Two hundred and thirty questionnaires were returned fully completed, giving a response rate of 59%. Fourteen statements regarding desirable support were rated with mean values > 6.00 and SD < 1.00 and considered as being the most important during the first year in the EMS. The important supports regarded; colleagues and work environment, management and organisation, experience-based knowledge, introduction period, practical support, and theoretical support. Most statements regarded culture and climate and the way the newcomers wanted to be treated.

**Conclusion:**

It was concluded that an important way to support newcomers in the EMS is to treat them ‘nice’. This can be achieved by creating an open climate and a welcoming culture where the new professionals feel trusted and treated with respect, created ways to work structurally, have applicable medical guidelines, and for newcomers to receive feedback on their actions.

## Introduction

Being new to a professional practice is a challenging period of time. New nurses often report feelings of stress and anxiety, and medical errors may be made due to inexperience [25]. Supporting new professionals during their first year seems to be key to strengthening self-confidence and reducing stress levels [22]. However, to our knowledge, there is a lack of knowledge about how to support new professionals in the emergency medical services (EMS).

## Background

The EMS has been described as extremely different compared to in-hospital environments. At hospital, teams are larger than in the EMS, and physicians, advanced equipment and patients’ medical history are often available [18]. In the EMS, a professional need to be prepared to care for patients in all aspects of life, with varying illnesses or injuries, sometimes in extreme environments, with limited resources and limited back-up. In previous research, some of the specific challenges of the EMS context have included the need to be flexible, having the ability to work independently, and being prepared for anything [19, 27]. These EMS-specific challenges may be even greater for a newcomer.

New professionals often enter a new professional practice with a feeling of being confused and inadequate. There is a period of transition where a new professional identity is formed [7]. The transition period is described as a multifaceted experience where, unfortunately, most emotions are negative, such as stress and anxiety [4, 22]. According to Benner [5] new professionals spend their first period of time in a new professional practice relying on written guidelines, and they focus on learning routines. However, routines may be difficult to learn in a practice like the EMS where professionals encounter situations that are highly varying and unpredictable, and the guidelines have been described as inadequate and often not applicable [14, 18].

Formal support models or mentorship programmes have been suggested to have positive effects on new professionals’ development, self-confidence and even the intention to stay within the organisation, according to in-hospital research [3, 22]. In contrast, lack of support for newcomers may lead to medical mistakes and lack of support may also increase the likelihood of these new professionals leaving the profession [10, 25].

In the EMS, due to its unpredictability and lack of resources, research regarding the effects of formal support for new professionals’ development, self-confidence and retention would be needed. However, to our knowledge little is known about what formal support new professionals in the EMS want and/or need for their professional development.

As a contribution to increasing the knowledge about how to support new professionals in the EMS, this study aims to describe what ambulance nurses consider to be important support during the first year in the EMS.

## Method

A cross-sectional study design with questionnaires was used.

### Participants and data collection

The level of formal education prior to working in the EMS differs around the world [13]. In Sweden, where this study was undertaken, the national requirements are that at least one of the team members in the ambulance must be a registered nurse, preferably with a one-year additional education leading to a specialist degree in prehospital emergency care [21]. This study involves nurses holding a specialist degree in prehospital emergency care and the participants will be referred to as ambulance nurses.

The study included all ambulance nurses that graduated during the years 2015 and 2016, from the 11 universities in Sweden that provide the one-year additional education to become a specialist nurse in prehospital emergency care. Home addresses of the ambulance nurses were obtained via registration offices at the 11 universities. A total of 396 participants were identified, and 395 letters and one email were sent in January 2018.

The letters contained information about the study, the questionnaire, a pre-paid return envelope and a personal code to a web version of the questionnaire in case the ambulance nurses preferred to answer the questionnaire electronically. The one email contained the same information about the study and a link to the questionnaire. A reminder was sent 3 weeks later to all non-responders. Five envelopes were returned due to being undeliverable and two questionnaires were returned with an explanation that the recipient had never worked in the EMS, leading to a total *n* = 389 eligible participants.

### Questionnaire

The statements in the questionnaire were developed and validated for content in a Delphi study by Hörberg et al. [17]. The questionnaire included eight demographic questions and 70 statements about support in the EMS, covering the following content areas: Practical support (14 statements), Theoretical support (nine statements), Support for theoretical knowledge (seven statements), Experience-based support (five statements), Support in terms of an introduction period (six statements), Support from colleagues and work environment (13 statements), Support from management and organisation (12 statements) and “Other” support (four statements).

The participants were asked to think about their own first year and grade each statement according to perceived importance on a seven-grade Likert type scale (1 = not important to 7 = very important).

### Statistics

Regardless of questionnaires being answered using the paper version or electronically, all data were registered in the survey tool software Survey&Report© version 4.2 (Artologic.net, Växjö, Sweden).

In agreement with Norman [23] the Likert scale type gradings were attributed as numbers, not labels, and treated as interval data. Descriptive statistics, frequencies, mean and standard deviation (SD) were computed. The dependent variables were found to be normally distributed and an independent t-test was used to compare means regarding, gender, level of prior EMS experience and geographic region. To explore whether there were any differences regarding prior EMS experience, the Benner [5] version of the skills acquisition theory was used as a basis for dichotomising the self-assessed years of experience into two groups; less experience (< 3 years of prior EMS experience) and experienced (> 3 years of prior EMS experience). Geographic region was calculated on the urban and rural group, where a difference in geography was notable. Professionals working in ambulances in or near a large city region have shorter transportation times to hospital than those in rural areas. In rural areas, the ambulance stations more often have a single ambulance and therefore the chances of acquiring an additional ambulance, if needed, is reduced compared to EMS in or near a large city region.

To determine the equality of variance of the measured groups, Levene’s Test of Equality of Variances was used. The level of statistical significance was set to *p* < 0.05. For data analysis, the statistical software package for Mac, SPSS version 24 (SPSS Inc., Chicago, IL, USA) was used.

## Ethical considerations

The study was approved by the regional ethics committee in Stockholm (2015–87 31/5). Information about the study was sent along with the questionnaire and it was highlighted that participation was voluntary. Confidentiality was guaranteed and participants were informed that they could leave the study at any time. Informed consent was considered achieved by answering the questionnaire.

## Result

Two hundred and thirty (*n* = 230) questionnaires were returned fully completed, giving a response rate of 59% (Table 1). One hundred and five participants identified themselves as men and 125 as women.

**Table 1:** Information on participants

Demographics	Number (percent)	
Eligible participants	389 (100%)	
Returned questionnaires	230 (59%)	
Demographic distribution of returned questionnaires	230 (100%)	
Gender		
Men (M)	105 (45%)	
Women (W)	125 (55%)	
Age		
20–30	60 (26.4%)	M:20/W:40
31–40	113 (48.9%)	M:61/W:52
41–50	44 (19.4%)	M:22/W:22
> 50	13 (5.3%)	M:2/W:11
Geographic region		
Urban*	39 (16.3%)	M:24/W:15
Middle range city	112 (49.3%)	M:55/W:57
Rural*	79 (34.4%)	M:26/W:53
Years of EMS experience		
< 1	10 (4.8%)	M:3/W:7
1–3	54 (23.8%)	M:19/W:35
3–5	94 (40.1%)	M:42/W:52
> 6	72 (31.3%)	M:41/W:31

*Urban was defined as large city regions, in Sweden these are; Stockholm, Gothenburg and Malmo. Rural was defined as geographic regions with < 5 people/km2, Swedish Board of Agriculture [28]

One hundred and seventy-four (76%) questionnaires were returned via mail and fifty-six (24%) were completed via the web version.

Twenty-eight of the 70 statements regarding desirable support in the EMS were rated with mean values > 6.00. In 14 of these statements, SD was < 1.00 (Table 2). These 14 support statements will henceforth be considered as being the most important during the first year in the EMS. Mean values of all 70 statements are presented in Appendix.

**Table 2:** Result of the 14 most important statements regarding support for new professionals in the EMS, mean value> 6.0 and SD < 1.0

*Area of content*			
Support in terms of:	Statement:	Mean	Std. Deviation
Introduction period	Have a structured introduction period	6,73	0,71
Colleagues and work environment	Get peer support debriefing in extreme situations	6,71	0,68
Colleagues and work environment	Have a trustworthy colleague	6,68	0,65
Theoretical support	Have access to applicable medical guidelines	6,68	0,69
Colleagues and work environment	Be respected and accepted by the colleagues at the ambulance station	6,59	0,68
Colleagues and work environment	There is an open climate at the ambulance station	6,56	0,68
Management and organisation	Trust in the ambulance station manager	6,50	0,77
Management and organisation	The organization is characterized by professionalism	6,50	0,82
Practical skills training	Practice methods to get a structured way to work (e.g. according to the ABCDE-principle)	6,50	0,89
Management and organisation	The organization is characterized by equally	6,50	0,90
Colleagues and work environment	Have an experienced colleague	6,47	0,89
Experience-based support	Participate in courses along with experienced colleagues	6,43	0,87
Experience-based support	Get feedback on the own actions from the receiving unit	6,40	0,97
Management and organisation	The organization provides time for professional development activities	6,20	0,95

The statistical significance (*p* < 0.05) of what ambulance nurses consider to be important support in the context of EMS based on gender, years of prior EMS experience, and geographic region is highlighted and presented in Table 3.

**Table 3:** Statistical significance in important support for new professionals in the EMS, based on Gender, Years of prior EMS experience and Geographic region

Statement	Gender			Years of EMS experience			Geographic region		
	Male	Female	*p*-value	< 3 yrs	> 3 yrs	p-value	Urban	Rural	*p*-value
Have a structured introduction period	6,67	6,68	0,246	6.75	6,72	0,752	6,77	6,68	0,579
Get peer support debriefing in extreme situations	6,70	6,73	0,716	6,70	6,72	0,891	6,59	6,82	0,108
Have a trustworthy colleague	6,55	6,79	0,007*	6,69	6,68	0,943	6,62	6,87	0,016*
Have access to applicable medical guidelines	6,59	6,76	0,063	6,72	6,67	0,624	6,31	6,75	0,027*
Be respected and accepted by the colleagues at the ambulance station	6,43	6,73	0,001*	6,53	6,61	0,447	6,41	6,75	0,019*
There is an open climate at the ambulance station	6,46	6,64	0,046*	6,67	6,51	0,086	6,41	6,65	0,137
Trust in the ambulance station manager	6.31	6,65	0,002*	6,63	6,45	0,073	6,36	6,58	0,151
The organization is characterized by professionalism	6,33	6,63	0,008*	6,52	6,49	0,819	6,13	6,68	0,013*
Practice methods to get a structured way to work (e.g. according to the ABCDE-principle)	6,36	6,62	0,035*	6,61	6,46	0,246	6,49	6,53	0,812
The organization is characterized by equally	6,38	6,60	0,076	6,52	6,49	0,871	6,15	6,73	0,010*
Have an experienced colleague	6,31	6,61	0,013*	6,67	6,40	0,008*	6,38	6,48	0,630
Participate in courses along with experienced colleagues	6,27	6,58	0,008*	6,44	6,43	0,977	6,38	6,57	0,228
Get feedback on the own actions from the receiving unit	6,31	6,48	0,200	6,39	6,41	0,895	6,36	6,52	0,457
The organization provides time for professional development activities	6,08	6,31	0,061	6,23	6,19	0,767	6,28	6,24	0,816

*p < 0.05

The mean values of the 14 most important support statements ranged from 6.73–6.20.

The important support statements concerned the following content areas: Support from colleagues and work environment, Support from management and organisation, Experience -based knowledge, Support in terms of an introduction period, Practical support, and Theoretical support. None of the 14 highest rated statements concerned support in terms of theoretical knowledge or ‘Other’ support. The mean values in all the 70 statements were higher in the women’s responses compared to the men’s, although not all were statistically significant.

### Support from colleagues and work environment

Five of the 14 most important support statements were about support from colleagues and the work environment: *Have an experienced colleague, Have a trustworthy colleague, Be respected and accepted by the colleagues at the ambulance station, There is an open climate at the ambulance station, Get peer support debriefing in extreme situations*. To have an experienced colleague was graded higher among women than men (*p* = 0.013) and by EMS professionals with less than 3 years of prior EMS experience compared to those with more than 3 years of experience (*p* = 0.008). To have a trustworthy colleague was graded higher among women than men (*p* = 0.007) and by those working in rural areas compared to those working in urban areas (*p* = 0.016). There was also a statistically significant difference in gender regarding being respected and accepted by colleagues, where women graded this higher than men (*p* = 0.001), and professionals working in rural areas graded it higher compared to those working in urban areas (*p* = 0.019). Women graded the support of having an open climate at the ambulance station higher than men (*p* = 0.046).

### Support from management and organisation

Four of the highest rated statements were about support from management and organisation, *Trust in the ambulance station manager, The organisation is characterised by professionalism, The organisation is characterised by equality* and *The organisation provides time for professional development activities.* Support in terms of trust the ambulance station manager was graded higher by women than men (*p* = 0.002), and that the organisation was characterised by professionalism was graded higher both by women compared to men (*p* = 0.008) and by professionals working in rural areas compared to those working in urban areas (*p* = 0.013). Professionals working in rural areas also graded support in terms of the organisation being characterised by equality higher than those working in urban areas (*p* = 0.010).

### Experience-based knowledge

In the content area; Experience-based knowledge, two statements were graded among the 14 most important: *Participate in courses with experienced colleagues* and *Get feedback one’s own actions from the receiving unit.* Regarding these two support statements, there was a statistically significant difference in support in terms of being able to participate in courses with experienced colleagues, where women graded this higher than men (*p* = 0.008).

### Support in terms of an introduction period

In this area of content one statement was considered among the most important: *Have a structured introduction period.* To have a structured introduction period was also graded the highest of all support statements in the questionnaire. There was no statistical significance in gender, years of prior experience or geographic region for this statement.

### Practical support and theoretical support

In the two content areas, Practical support and Theoretical support, only one statement each was graded among the most important. *Practice methods to develop a structured way to work (*e.g. *according to the ABCDE principle)* and *Have access to applicable medical guidelines.* To practice methods to develop a structured way to work was graded higher among women than men (*p* = 0.035). To have applicable medical guidelines was graded higher by those working in rural areas compared to those working in urban areas (*p* = 0.027).

## Discussion

Equality, acceptance, professionalism, trust and respect seemed to be aspects of support that was considered to be important. Since most of the 14 statements in the results were about the climate in the organisation, it seems that the way new professionals are treated and welcomed into the EMS practice are more important to the professionals themselves than for example to have practical skills training.

The importance of being treated as a respected member of the new community, i.e. being part of a professional practice, has other important implications than merely for the professionals to feel welcome. Lave and Wenger describe participation and sense of belonging as key to development of new knowledge. It is by being a legitimate participant in a community that people learn and the community develops [29]. In a previous study about the experience of being new to the EMS, professionals described a macho and unsupportive culture and an experience of being bullied into the new profession [18]. Unfortunately, similar experiences have been described in other contexts where one barrier to support for new professionals is the attitudes of the experienced professionals [11]. Experienced professionals describe their new colleagues as incompetent, not trustworthy, and as unable to assess patients correctly or fast enough [11, 16]. Professionals who did not get support themselves when they were new perceive supporting new colleagues as unnecessary, which may be reflected in the way they treat their new colleagues [11]. Unsupportive behaviours by experienced peers such as rude remarks and unjust criticism may lead to new professionals’ confidence being undermined, interfering with professional development [24]. It seems important that organisation managers are aware of the silent cultures and climate in their own organisations and they should actively work to implement a welcoming atmosphere on all levels, where the new professionals are treated with respect. Ideal structures for learning in practice can be created through a common ground, mutual trust, pride in the purpose of the work and a shared set of ideals [29].

In traditional EMS training, skills training and training via simulation have been suggested to increase professionals’ competence in a patient-safe manner [1]. Another study by Knox et al. [20] concluded that, according to the professionals, the most important support for continuous professional competence is practical training scenarios, simulation practice and practice with manikins. However, the above-mentioned research mainly involved experienced professionals. We do not believe our study results diminish the evidence that suggests the positive effects of practice through simulation. However, even though *Practice through simulation* was considered important in this study (Appendix) it was graded as less important than for example an *Open climate at the ambulance station* or *Have a trustworthy colleague*. These findings suggest that new, inexperienced professionals and experienced professionals may differ in how their professional development should be supported.

This result adds to the field of knowledge about the development of EMS professionals’ competence in that other aspects than merely simulation and practical skills training are important.

Of the 14 most important support statements, *A structured introduction period, Support in creating ways to work structurally* and *Have applicable medical guidelines* may be considered to reflect a novice professional’s desire to create a sense of control. In the Dreyfus stage model of skills acquisition by Benner [5] novice nurses are described as lacking experience and therefore they rely on rules and written guidelines to guide their performance. Our study results support the notion that new professionals need applicable guidelines during the first year of practice.

Furthermore, to *Get feedback on one’s own actions from the receiving unit* was among the highest graded support statements. When new and inexperienced, feedback can be seen as a way for the new professional to get confirmation on cognitive and technical performance, and when received from a credible source and constructively, feedback can change clinical performance and promote professional development [2]. The need and perceived benefits of feedback in the EMS have been stressed before [8, 30] but unfortunately it seems difficult to implement on a general level [8]. The awareness of the positive impact of feedback on patient safety and professional development may need to be further highlighted.

The mean values of all the 70 statements were higher in the women’s responses compared to the men’s and although not all mean differences were statistically significant, this general difference between the genders was an interesting result. Even though differences between genders have been described as decreased during the last years, traits such as expressivity, warmth and concern for the welfare of others are still considered female traits while competence, instrumentality and independence are considered male [12]. To the best of our knowledge there is a lack of research in the EMS that focuses on gender. One study by Blau et al. [6] explored differences in job satisfaction and emotional labour between men and women in the EMS. However, in their study no statistical significance in differences between gender was found. The reason for the statistical difference shown in our study may be difficult to assess. One reason could be that women are believed to have higher levels of concern about risks than men [15]. Being more concerned about possible risk may lead to higher grading in considered importance of support. Another possible reason for the gender difference in this study could be that men and women express their opinions differently. More research addressing gender differences in the EMS is needed to determine whether or not there is actually a difference in opinion or a difference in how opinions are expressed between genders in the EMS.

In the EMS, experience has been described as one of the most important tools for managing challenging situations. Surprisingly, only in one of the 14 statements, to *Have an experienced colleague*, was there a significant difference between professionals with more experience (> 3 years) and those with less experience (< 3 years). However, two of the 14 most important supports were about learning from the experience of others, *Participate in courses with experienced colleagues* and *Have an experienced colleague.* This may suggest that one’s own experience may have little impact on the perceived importance of support during the first year and that learning from the experience of others may be an important way of supporting new professionals.

In formal education, content is often decontextualised and taught independently of practice. It is then assumed that the educated professional will be able to perform appropriately in any situation. According to Harenčárová [16], 60% of the EMS professionals reported uncertainty related to inadequate understanding of the situation. In the EMS, the encountered situations can be extremely varied and knowing how to handle everything is a challenge that causes stress and anxiety for many new EMS professionals [18]. Adding to the challenge of extremely varied situations and high demands on knowledge, is the argument that professional knowledge cannot be acquired just through formal education or by doing [9]. The considered importance of working with an experienced colleague and participating in courses with experienced colleagues indicate a need to learn from colleagues’ experiences and “war stories”. Working with an experienced colleague may also create a sense of security that may further facilitate development. Reflection in and on action has been suggested to be a key to the translation of formal knowledge acquired from education to professional knowledge and skills [9, 26]. Reflective ability and organised reflection seminars in the EMS may also lead to an increased sense of security and competence in the professionals and may thereby increase patient safe actions [30]. However, a prerequisite for the ability to reflect is trusting other colleagues and feeling secure in the new environment.

## Conclusion

Organisations can support new professionals by creating an open climate and a welcoming culture where the new professionals feel trusted and treated with respect in their new EMS community. Creating an open climate in the organisation may also facilitate reflection on and in action, together with experienced colleagues. Working with an experienced colleague may be a way to bolster the new professional’s sense of belonging and participation in the new community.

New professionals also strive to create a sense of control and also like to receive confirmation that they have made the right decisions. A structured introduction period, support in creating ways to work structurally, having applicable medical guidelines, and receiving feedback on actions may further support new professionals.

Future studies are needed on formal support with a focus on culture and climate within an organisation. A next step could be to implement and evaluate the effects of formal support in the EMS, based on the results of this study.

### Limitations

There are some limitations that need to be considered in this study. First, 41% of the eligible participants did not participate and there is no way of knowing why some of the respondents chose not to participate due to the study design. However, the non-response analysis showed no explicatory difference concerning gender or examined university between respondents and non-respondents. Cross sectional studies give a snapshot of a reality and can therefore be difficult to reproduce. It may also be difficult to assess whether any difference in result in a reproduced cross sectional study relate to attitudes being changed over time or due to other participants agreeing to participate. With cross sectional survey studies, there is a risk of response bias such as the risk of participants not wanting to give socially unacceptable or embarrassing answers. Even though this study was not considered to measure and socially unacceptable issues, we cannot with certainty know that the participants did not experience the statements as such.

Another possible limitation is that this study has a Swedish perspective and the EMS professionals were ambulance nurses. We acknowledge that ambulance nurse is not a standard level of education around the world and this may affect generalizability in other study settings. The questionnaire used in this study was study specific, and developed out of a prior, published Delphi study [17, 18]. The content validity of the statements used was thereby assessed by an expert group and validated for purpose and that the questions could be understood. However, further validation need to be performed to use to questionnaire in a larger and/or international setting. Despite these limitations, we believe this study give a picture of what ambulance nurses in Sweden consider to be important support.

## Appendix

Please insert Table 4 here.

**Table 4:** Result of a cross-sectional survey study about important support during the first year in the EMS

**Statements**		**Mean**	**Std. Deviation**
**Support from practical skills exercises**			
**9.1**	**Practice methods to get a structured way to work (e.g. according to the ABCDE-principle)**	**6,50**	**0,89**
9.2	Practice through simulation	5,97	1,24
9.3	Practice situations that occur rarely	5,74	1,34
9.4	Practice situations that occur frequently	5,51	1,33
9.5	Practice in collaboration with police and rescue service	5,67	1,23
9.6	Practice ways to lead the work at the scene of an accident	6,02	1,12
9.7	Practice with the radio communication equipment	5,84	1,29
9.8	Practice with the medical equipment in the ambulance	6,13	1,25
9.9	Practice techniques for immobilization	5,99	1,12
9.10	Practice techniques for removing people from vehicles	5,66	1,37
9.11	Practice techniques to maneuver the stretcher	5,24	1,58
9.12	Driving and parking exercises	5,41	1,43
9.13	Have practical skills tests	5,47	1,50
9.14	Get a structured run-through of the medications used in the EMS	6,27	1,10
**Support from theoretical knowledge**			
10.1	Be able to visit and auscultate at different intra-hospital wards	4,89	1,73
10.2	Have access to lectures on medical conditions in adults	5,71	1,25
10.3	Have access to lectures on medical conditions in children	6,07	1,09
10.4	Have access to lectures on child birth	5,80	1,18
10.5	Have access to lectures on how to lead the work at the scene of an accident	5,60	1,21
10.6	Get access to lectures about psychiatric conditions	5,37	1,38
10.7	Get a structured run-through of the EMS medical guidelines	6,21	1,02
10.8	Get access to concept educations such as AMLS, PHTLS, PS, PEPP	6,08	1,23
10.9	Have written tests on theoretical knowledge	5,20	1,48
**Theoretical support**			
**11.1**	**Have access to applicable medical guidelines**	**6,68**	**0,69**
11.2	Have access to written ethical guidelines	5,03	1,45
11.3	Have access to written guidelines on when and how to contact a physician	5,83	1,32
11.4	Have access to written guidelines about how to report deviations	5,27	1,37
11.5	Have access to written guidelines regarding how to manage conflicts	4,82	1,44
11.6	Have access to internet-based instruction films on the ambulance’s technical equipment	5,29	1,49
11.7	Have access to instruction films about how to realign a fracture	5,26	1,48
**Support for experience-based knowledge**			
**12.1**	**Participate in courses along with experienced colleagues**	**6,43**	**0,87**
12.2	Participate in group discussions about authentic patient situations	6,27	1,04
12.3	Participate in group discussions about ethics	5,68	1,41
12.4	Participate in group discussions about threats and violence	6,07	1,12
**12.5**	**Get feedback on the own actions from the receiving unit**	**6,40**	**0,97**
**Support from an introduction period**			
**13.1**	**Have a structured introduction period**	**6,73**	**0,71**
13.2	Have an individually fitted introduction period	6,14	1,20
13.3	Have a supervisor with formal supervisor competence during the introduktion period	5,56	1,55
13.4	Work with the same supervisor during the introduction period	5,27	1,47
13.5	Work with the same ambulance team (supervisor and his/her colleague) during the introduction period	4,71	1,65
13.6	Get regular feedback on the own development during an introduction period	6,28	1,01
**Support from colleagues and work environment**			
**14.1**	**Have an experienced colleague**	**6,47**	**0,89**
14.2	Work with the same colleague during the first year	3,21	1,65
14.3	Work with another RN	5,38	1,68
**14.4**	**Have a trustworthy colleague**	**6,68**	**0,65**
**14.5**	**Be respected and accepted by the colleagues at the ambulance station**	**6,59**	**0,68**
**14.6**	**There is an open climate at the ambulance station**	**6,56**	**0,68**
**14.7**	**Get peer support debriefing in extreme situations**	**6,71**	**0,68**
14.8	Have one person in the organization to contact with logistics questions during off-hour	5,89	1,23
14.9	Have a mentor to support personal development	5,30	1,49
14.10	Have a mentor to support professional development	5,39	1,42
14.11	Have a mentor to talk to about conflicts	5,37	1,43
14.12	Have a mentor to contact about practical issues	5,18	1,50
14.13	Have a mentor to contact about routines	5,07	1,53
**Support from management and organization**			
15.1	Get feedback on the own professional development from the ambulance station manager	5,77	1,23
15.2	Get feedback on the own professional development from the organization director	3,99	1,73
**15.3**	**Trust in the ambulance station manager**	**6,50**	**0,77**
15.4	Trust in the organization director	5,35	1,53
15.5	Have confidence in the organization	6,18	1,01
15.6	The organization is characterized by ethical considerations	6,13	1,10
**15.7**	**The organization is characterized by professionalism**	**6,50**	**0,82**
**15.8**	**The organization is characterized by equally**	**6,50**	**0,90**
15.9	The organization has clear competence descriptions of what is expected of each role in the team	6,14	1,07
**15.10**	**The organization provides time for professional development activities**	**6,20**	**0,95**
15.11	The organization accepts that new professionals need more time to perform patient assessments	6,20	1,11
15.12	The dispatch center accepts that new professionals need more time to perform patient assessments	5,52	1,69
**Other support**			
16.1	Being exempt from life-threatening assignments with the highest level of priority	1,76	1,44
16.2	Receiving an extra unit when being given life-threatening assignments with the highest level of priority	2,67	1,78
16.3	Being exempt from introducing new colleagues	5,82	1,66
16.4	Have access to an interpreter service	4,63	1,82

Participants *n* = 230, response rate 59%. Highest rated statements in bold
